# The deaths of invisible people. A literature review

**DOI:** 10.1192/j.eurpsy.2024.1639

**Published:** 2024-08-27

**Authors:** M. I. Santana Ortiz, N. Molina Pérez, J. Pereira López

**Affiliations:** Psychiatry, University Hospital of Gran Canaria Dr. Negrin, Las Palmas de Gran Canaria, Spain

## Abstract

**Introduction:**

Suicide is a serious public health problem. Each year it is estimated that it causes almost one million deaths worldwide, much more than those caused by war or homicide. These deaths are also devastating, affecting not only the person who commits them, but also his or her environment (family, friends, professionals involved, etc.) and society as a whole. The risk and protective factors for suicide are well known in the literature, which gives rise to the estimation of possible high-risk groups according to their characteristics, especially when risk factors are added, protective factors are reduced, and unfavorable life circumstances are present. Among these groups with greater vulnerability to suicidal behavior are homeless people with severe mental disorders, who are unfortunately little visible in society and in the investigation.

**Objectives:**

The aim of this paper is to review the current state of the question of suicide in homeless people with severe mental disorders.

**Methods:**

Review of the international scientific literature on the issue published in the last twenty years.

**Results:**

The few studies available conclude the higher prevalence of suicidal behavior in homeless people with severe mental disorders compared to the general population, which has not been translated into the development of specific care and prevention plans and programs.

**Conclusions:**

It is considered essential to expand investigation in this field, which will be very useful to lay the foundations for the development of guidelines, plans and specific programs, and to know the evidence about them.

**Disclosure of Interest:**

None Declared

